# Determination of Trace Amounts of Hydrofluoric Acid in Non-Aqueous Solutions by the Coulometric Titration Method

**DOI:** 10.3390/s18124439

**Published:** 2018-12-15

**Authors:** Cairui Huang, Congcong Shen, Ling Jin, Hongwei Cai

**Affiliations:** Department of Chemistry, School of Chemistry, Chemical Engineering and Life Science, Wuhan University of Technology, Wuhan 430070, China; huangcairui@whut.edu.cn (C.H.); 1049731701231@whut.edu.cn (C.S.); lingjin@whut.edu.cn (L.J.)

**Keywords:** hydrofluoric acid, non-aqueous solution, coulometric titration, acid–base coulometric autotitrator

## Abstract

For monitoring of trace amounts of hydrofluoric acid in the organic fluorine chemical industry, a facile method for determination of the hydrofluoric acid in an ethanol solution of lithium chloride, by coulometric titration, was proposed. Relying on homemade acid–base coulometric autotitrator, the electrolyte was 0.50 mol·L^−1^ LiCl ethanol solution and the constant current intensity was 0.2–2 mA. As for the working electrode pair, a platinum plate was used as a working electrode, and a platinum wire was used as an auxiliary electrode. The indicating electrode was the pH composite glass electrode and the titration endpoint was pH 5.50. The results showed that the relative standard deviation was below 2.0%, as the content of the hydrofluoric acid was between 2 μg to 100 μg. The recovery rate was 99.0–102.0%. This proposed route has the advantages of simplicity, convenience, quickness, accuracy, and automation, which can be applied to the accurate determination of trace amounts of hydrofluoric acid, in non-aqueous solutions.

## 1. Introduction

Fluorine gas and anhydrous hydrogen fluoride are important raw materials in the organic fluorine chemical industry. They are used to prepare many fluorine compounds with different types and functions. The products are widely applied in the fields of chemical industry, machinery, electronics, energy, metallurgy, medicine, pesticides, etc. [1,2,3,4,5,6,7]. However, due to the hydrolysis of fluorine gas or the residue within the productive process, the inevitable, existing hydrogen fluoride will affect the quality of the organic fluoride products. For example, 1,1,1,3,3-pentafluoropropane (HFC-245fa), which can be substituted for refrigerants, detergents, and foaming agents, is generally fluorinated by the reaction between anhydrous hydrogen fluoride and the pentachloropropane in carbon tetrachloride. The remaining hydrogen fluoride must be removed [8]. In the process of preparing trifluoroacetic acid by electrofluorination, electrochemical fluorination of acetic acid or acetic anhydride, with hydrofluoric acid, is carried out, and then is hydrolyzed to form trifluoroacetic acid. The by-product—hydrofluoric acid—accompanies the hydrolysis process, which also affects the quality of the product [9]. Under normal circumstances, the content of hydrofluoric acid in ordinary fluoride products is required to be less than 0.010%, and the superior grade fluoride products is even required to be less than 0.001%. All these demand a rapid and accurate method for the determination of the trace hydrofluoric acid in a non-aqueous solution.

At present, the traditional acid–base titration and potentiometric titration are generally used to determine hydrofluoric acid [10,11]. Although the methods are simple and convenient, they are not suitable for the determination of the trace hydrofluoric acid. Ion chromatography, fluorescence analysis, infrared spectroscopy, etc., have also been proposed [12,13,14]. However, those applications are limited by the expensive equipment. Therefore, it is necessary to propose a simple, rapid, and accurate method for the determination of the trace hydrofluoric acid in non-aqueous solution.

Coulometric titration is based on a quantitative reaction between the investigated substance and the titrant generated from the electrolyzation of the solution, under a constant current. The content of measured substance can be obtained directly or indirectly, by the electric quantity. It is the most accurate macro analysis method, so far, and it is also a sensitive method for the accurate determination of trace substances. It has the advantages of simplicity, convenience, quickness, accuracy, and automation [15]. The determination of acid and alkali in non-aqueous solutions, through coulometric titration, has been reported in a previous study. Mihajlović [16] proposed that the alkaline ions (m-cresolate ions) could be electrogenerated to determine acid in a γ-butyrolactone solution, by using m-cresol as the cathodic depolarizing agent, and tetrabutylammonium perchlorate as the supporting electrolyte. Johansson [17] titrated acids by generating OH^−^ in isopropanol or a mixture of isopropanol and methylacetone, which contained a trace of water. Champion [18] determined organic acids by a coulometric generation of the base, at a platinum cathode, in tetrahydrofuran containing about 0.2% of water. Gonzaga [19] used lithium chloride as a supporting electrolyte to determine the acid content of the ethanol fuel. Mihajlović [20] summarized the studies of certain strong acid or base, which can be generated through electro-oxidation or a reduction in non-aqueous solvents. These compounds were used as anodic or cathodic depolarizers for coulometric titrations of acids, bases, and salts. All the above work proved that the coulometric titration has a high accuracy in the determination of acid–base substances in non-aqueous solution. A method for the determination of trace amounts of hydrofluoric acid in an ethanol solution of lithium chloride, by coulometric titration, is proposed in this paper. Satisfactory results are obtained, which lays a good foundation for further in-depth study.

## 2. Materials and Methods

### 2.1. Reagents and Chemicals

0.10 mol·L^−1^ NaOH ethanol solution: The accurate concentration was determined by using benzoic acid as the reference substance and bromothymol blue as the indicator.

0.10 mol·L^−1^ HF ethanol solution: The accurate concentration was determined by an NaOH ethanol solution and bromothymol blue as the indicator.

2–100 μg·mL^−1^ HF solution: The solution was diluted by 0.10 mol·L^−1^ HF ethanol solution step by step, the diluent was anhydrous ethanol.

Anhydrous methanol, anhydrous ethanol, isopropanol, acetone: AR, treated with 3A molecular sieve, to remove the residue of water before use.

0.50 mol·L^−1^ LiCl ethanol solution: About 10.6 g lithium chloride was weighed and dissolved in 500 mL anhydrous ethanol.

All other chemicals were of the analytical grade, or above.

### 2.2. Instrumentation

Homemade automatic acid–base coulometric titration instrument, platinum electrode (5 mm × 2 mm × 0.1 mm, Jiangsu Analysis Instrument Factory, Jiangsu, China); platinum wire auxiliary electrode (Φ 1 mm × 120 mm, Jiangsu Analysis Instrument Factory, Jiangsu, China); pH composite glass electrode (InLab Expert Pro, METTLER TOLEDO, Zurich, Switzerland).

**Figure 1:**
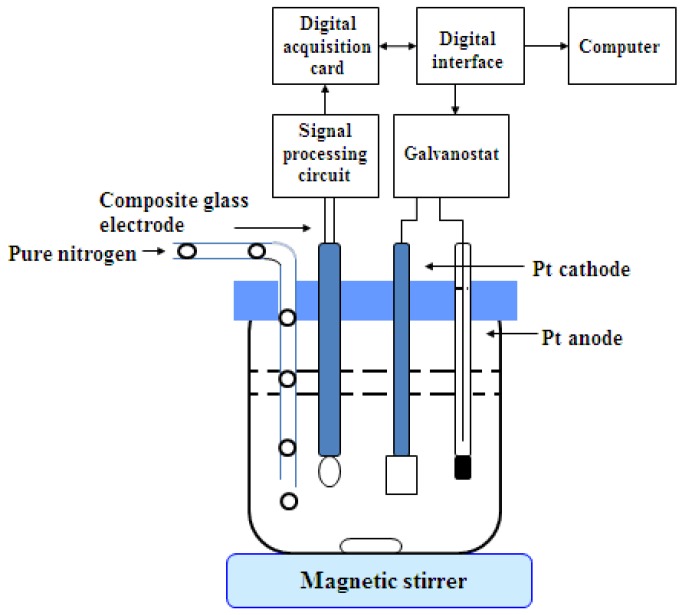
Acid–base coulometric autotitrator of constant current.

The schematic diagram of the automatic acid–base coulometric titration instrument is shown in Figure 1. It consisted of three parts—the electrolysis loop system, the indicating loop system, and the computer control system. The electrolysis loop system consisted of a digital constant current source, a platinum working electrode, and a platinum wire auxiliary electrode. The platinum wire auxiliary electrode was mounted in a glass sleeve and connected with the working electrode, through a ceramic sand core. The indicating loop system was composed of pH composite glass electrode, a signal conditioning circuit, and a data acquisition card NI6009. The signal conditioning circuit was used for amplification, filtering, and impedance conversion. The data acquisition card completed data acquisition, A/D conversion, and uploads to the computer, through an USB interface. The computer control system relied on a desktop computer and used LabView as a software development platform to realize the system parameter settings, pH conversion, electrolysis loop control, electrolysis timing, result calculations, and the output.

### 2.3. Determination Method

About 50 mL 0.50 mol·L^−1^ LiCl ethanol solution was prepared in a 100 mL PTFE beaker. Platinum electrode, platinum wire auxiliary electrode (filled with 0.50 mol·L^−1^ LiCl ethanol solution), and pH composite glass electrode were inserted into the solution.

A certain amount of the HF ethanol solution was accurately pipetted into the above solution, which was pre-deaerated by nitrogen and pre-titrated. The electrolysis was carried out on an automatic acid–base coulometric titration instrument. Instruction to stop the electrolysis would be sent to the electrolysis loop system, from the computer control system, when the titration endpoint was reached. The electrolysis process would be stopped, immediately, and the results were output automatically. The content of the HF was calculated using a constant current and electrolysis time. The corresponding formula is presented as follows:

$$\rho_{\mathrm{HF}}=\frac{\frac{It}{F}\times M\times 10^{3}}{V_{S}}$$

where *I* (mA) is the constant current, *t* (s) is the electrolysis time, *F* (96,500 C·mol^−1^) is the Faraday’s constant, *M* is the molar mass of the HF, *V*s (mL) is the volume of the added HF ethanol solution, *ρ*_HF_ (μg·mL^−1^) is the mass of the HF in the HF ethanol solution.

## 3. Results and Discussion

### 3.1. Selection of Organic Solvents

The ideal non-aqueous solvent for coulometric titration should have a high solubility for supporting the electrolyte and the conductivity of the resulting electrolyte solution should be high, which ensures that large currents can pass through the solution. Here, anhydrous methanol, anhydrous ethanol, isopropanol, and acetone were investigated as potential solvents. It was found that the current efficiency of acetone and isopropanol was less than 100%, and the maximum current intensity was less than 1 mA. In anhydrous methanol and anhydrous ethanol, the current intensity was very stable, the maximum value reached 3 mA, and the current efficiency was close to 100%. Table 1 shows the determination of hydrofluoric acid, with a known content, using an LiCl solution of different solvents.

Table 1 shows that the precision and accuracy of the determination of HF in the anhydrous ethanol were higher than those in the anhydrous methanol. Furthermore, compared with anhydrous methanol, anhydrous ethanol was less harmful and more environment-friendly. So, anhydrous ethanol was chosen as the organic solvent in this study.

### 3.2. Selection of Supporting Electrolytes and their Concentrations

Lithium chloride, sodium perchlorate, tetraethyl ammonium bromide, and tetraethyl ammonium perchlorate were selected as the supporting electrolytes. Their current efficiencies were investigated during electrolysis. The results showed that a yellow deposit was formed on the anode when tetraethyl ammonium bromide or tetraethyl ammonium perchlorate were used, which caused the continuous drop of current intensity. The current efficiency reached 100% when lithium chloride or sodium perchlorate was used. As sodium perchlorate is a dangerous strong oxidant, lithium chloride was chosen as the supporting electrolyte. Table 2 shows the determination of hydrofluoric acid in LiCl solutions, with different concentrations. When the concentration of lithium chloride reached 0.50 mol·L^−1^ and above, the precision and accuracy were below 0.50%. Therefore, 0.50 mol·L^−1^ LiCl ethanol solution was selected as the supporting electrolyte solution.

### 3.3. Selection of the Indicating Electrode Couples

Indicating electrode couples are very important in coulometric titration. They should have a quick response to the change of H^+^ activity and an obvious titration jump of the pH. The pH composite glass electrode (CGE) and the glass electrode (GE)-saturated calomel electrode (SCE) were investigated. It was found that their accuracy and precision met the experimental requirements. Since both electrodes had similar characteristics, the pH composite glass electrode was selected as the indicating electrode, in this study, due to its simplicity.

### 3.4. Selection of the Titration End-Point

**Figure 2:**
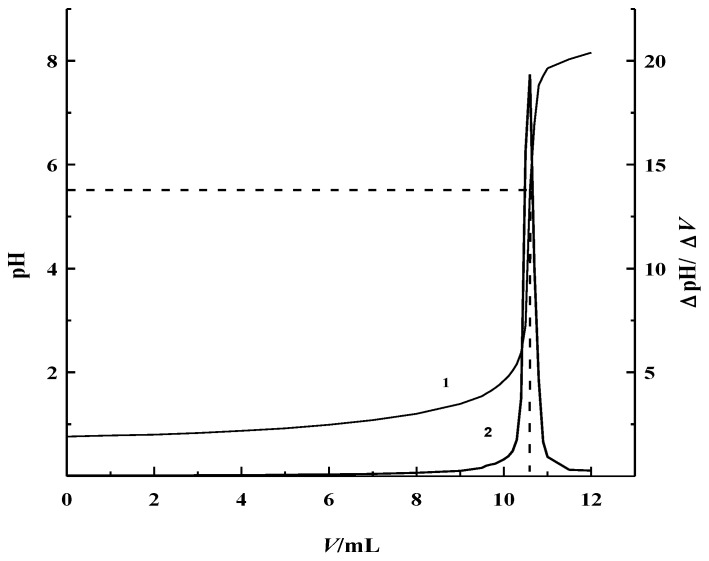
0.10 mol·L^−1^ HF potentiometric titration curve. (1. pH-*V*; 2. ΔpH/ΔV-*V*).

Certain amount of 0.10 mol·L^−1^ HF ethanol solution was placed into a 100 mL PTFE beaker and potentiometric titration was carried out with 0.10 mol·L^−1^ NaOH ethanol solution. Figure 2 shows the potentiometric titration curve of 0.10 mol·L^−1^ HF ethanol solution. It clearly shows that the ranges of titration jump was around pH 3.0–7.0. Its maximum jump was determined to be around pH 5.50, through its first order differential curve. Thus, pH 5.50 was used as the titration end-point of the coulometric titration for the determination of trace hydrofluoric acid, in a non-aqueous solution.

### 3.5. Pre-Titration

In order to eliminate the hysteresis effect of the pH composite electrode, in the process of electrolysis, pre-titration must be carried out before the determination process. A small and unmeasured amount of the HF ethanol solution was injected into the 0.50 mol·L^−1^ LiCl ethanol solution, and it was pre-titrated to the end-point. The solution of the HF sample was then added and the instrument was set to titrate automatically.

### 3.6. Results of Determination

Based on the investigations above, the optimized experimental conditions were obtained. The electrolyte was 0.50 mol·L^−1^ LiCl ethanol solution. The constant current intensity was 0.2–2 mA. The working electrode pair was a platinum working electrode and a platinum wire auxiliary electrode. The indicating electrode was a pH composite glass electrode and the titration end-point was pH 5.50.

**Figure 3:**
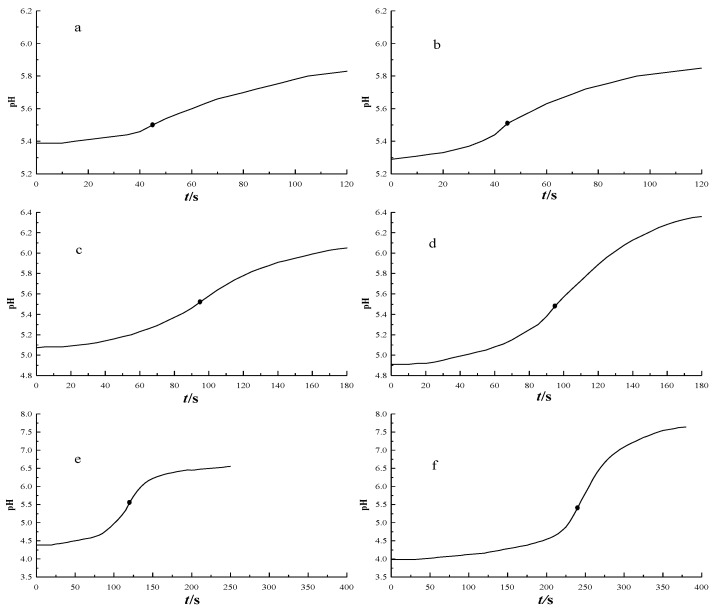
Coulometric titration curves of the hydrofluoric acid with different contents. vs. = 1.00 mL. (**a**) *ρ*_HF_ = 1.995 μg·mL^−1^, *I* = 0.201 mA; (**b**) *ρ*_HF_ = 4.966 μg·mL^−1^, *I* = 0.507 mA; (**c**) *ρ*_HF_ = 9.931 μg·mL^−1^, *I* = 0.507 mA; (**d**) *ρ*_HF_ = 19.86 μg·mL^−1^, *I* = 1.005 mA; (**e**) *ρ*_HF_ = 50.49 μg·mL^−1^, *I* = 2.004 mA; and (**f**) *ρ*_HF_ = 101.0 μg·mL^−1^, *I* = 2.004 mA.

Table 3 shows the determination results of the HF ethanol solution, with a different HF content. The accuracy and precision of the results were below 1.0%, when the HF content was 10 μg and above. Although the accuracy and precision of the determination of the HF content around 2 μg were slightly lower than 2.0%, it could still meet the actual measurement requirements, at such a low HF content. The corresponding coulometric titration curves are shown in Figure 3.

When the content of the hydrofluoric acid was too low, the decrease range of the pH of the solution became smaller, after pre-titration. When 2 μg was added, the pH only decreased from 5.5 to 5.4, and the relative standard deviation of the results reached 1.12%, and the relative error reached 1.90%. If 2.0% was used as a benchmark, the detection limit of the coulometric titrator was 2 μg.

### 3.7. Recovery

Table 4 shows the recovery after adding different contents of HF in the HF ethanol solution. The recovery rates were between 99.0–102.0%, which shows that the proposed method had no systematic errors and the determination was accurate and reliable.

## 4. Conclusions

Coulometric titration was adopted to determine trace amounts of HF in non-aqueous solution. It could be accomplished within 5 min, the precision and accuracy were below 2.0%, and the titration end-point could be detected automatically. It has the advantages of simplicity, convenience, quickness, accuracy, and automation. Compared with the traditional acid–base titration and potentiometric titration, the analysis time was notably shortened. As the titrant was produced through electrolysis, the preparation, standardization, and storage of the standard solution were avoided, and the endpoint could be automatically determined. Therefore, the results were more objective and accurate. Compared with ion chromatography, fluorescence analysis, and infrared spectroscopy, the experimental device was simpler, the operation was more convenient, the endpoint could be automatically judged, the influence factors were fewer, and the precision was higher.

Further applications for the determination of trace HF in non-aqueous solutions will be studied in-depth and reported in the follow-up work.

## Figures and Tables

**Table 1:** Determination of the presence of HF with different solvents (*n* = 5, vs. = 1.00 mL).

Solvents	Concentration of HF Solution (μg·mL^−1^)	Current (mA)	Time (s)	Determination Results (μg·mL^−1^)	*E*r (%)	RSD (%)
Acetone	50.35	‒ *	‒	‒	‒	‒
Isopropanol	50.35	0.502	472.98	49.23	2.22	1.89
Methanol	50.35	2.005	120.2	49.97	0.75	0.73
Ethanol	50.35	2.005	121.48	50.51	0.32	0.39

* When acetone was used as an organic solvent, it could not be determined because the current intensity was too small.

**Table 2:** Selection of the concentration of LiCl supporting electrolyte (*n* = 5, *I* = 2.005 mA, vs. = 1.00 mL).

Concentration of LiCl (mol·L^−1^)	Concentration of HF Solution (μg·mL^−1^)	Time (s)	Determination Results (μg·mL^−1^)	*E*r (%)	RSD (%)
0.10	50.35	120.14	49.95	0.79	1.03
0.30	50.35	120.43	50.07	0.56	0.66
0.50	50.35	121.48	50.51	0.32	0.39
0.80	50.35	121.40	50.47	0.24	0.43
1.0	50.35	121.56	50.54	0.38	0.42

**Table 3:** Determination results of the different HF content (*n* = 5, vs. = 1.00 mL).

Concentration of HF Solution (μg·mL^−1^)	Current (mA)	Time (s)	Determination Results (μg·mL^−1^)	*E*r (%)	RSD (%)
1.995	0.201	46.95	1.957	1.9	1.12
4.966	0.507	46.37	4.875	1.83	1.07
9.931	0.507	95.12	10	0.69	0.72
19.86	1.005	94.63	19.72	0.7	0.57
50.49	2.004	121.94	50.67	0.36	0.4
101	2.004	244.02	101.4	0.4	0.44

**Table 4:** Determination results of recovery (*n* = 5, vs. = 1.00 mL).

Measured (μg·mL^−1^)	Amount-Added (μg·mL^−1^)	Current (mA)	Time (s)	Amount-in Total (μg·mL^−1^)	Recovery (%)
9.543	4.828	0.505	138.09	14.46	101.8
	9.657	0.505	183.16	19.18	99.8
18.80	9.657	1.001	137.45	28.53	100.8
	19.31	1.001	182.79	37.94	99.1
49.72	25.03	2.010	179.52	74.82	100.3
	50.07	2.010	241.13	100.5	101.4
